# Supplementary material for: Smooth Interpolating Curves with Local Control and Monotone Alternating Curvature
Source: Comput Graph Forum. 2022 Oct 6;41(5):25–38. doi: 10.1111/cgf.14600 (PMC9827861; doi:10.1111/cgf.14600)
Supplement: Supplementary file 1 — Supplement Material [file CGF-41-25-s001.zip › Local-Smooth-Interpolating-MonoCurvature/extern/clothoids/submodules/quarticRootsFlocke/docs/api-cpp/typedef_a00023_1a1ac247f8669dde00caaec7b49131507f.html]

Typedef PolynomialRoots::complexType — Quartic Roots v1.0.0

### Navigation

- index
- toc
- next
- previous
- Quartic Roots »
- C++ API »
- Typedef PolynomialRoots::complexType

# Typedef PolynomialRoots::complexType¶

- Defined in File PolynomialRoots-Utils.hh

## Typedef Documentation¶

typedef std::complex<valueType> PolynomialRoots::complexType¶

### Quick search

### Table of Contents

- Quartic Roots Solver
- C++ API

«
hide menu

menu
sidebar
»

### Navigation

- index
- toc
- next
- previous
- Quartic Roots »
- C++ API »
- Typedef PolynomialRoots::complexType

© Copyright 2021, Enrico Bertolazzi.
Created using Sphinx 4.2.0.
